# Women’s Empowerment and Associated Factors in Kinshasa, Democratic Republic of Congo: A Secondary Data Analysis of the Performance Monitoring Assessment Survey

**DOI:** 10.3390/ijerph21070943

**Published:** 2024-07-19

**Authors:** Annick Makongote, Branly Mbunga, Pierre Akilimali, Sofia Castro Lopes, Dieudonné Mpunga

**Affiliations:** 1 Community Heath Master Program, School of Public Health, University of Kinshasa, Kinshasa P.O. Box 11850, Democratic Republic of the Congo; 2 Department of Nutrition, School of Public Health, University of Kinshasa, Kinshasa P.O. Box 11850, Democratic Republic of the Congopierre.akilimali@unikin.ac.cd (P.A.); 3 Patrick Kayembe Research Centre, School of Public Health, University of Kinshasa, Kinshasa P.O. Box 11850, Democratic Republic of the Congo; 4 Division of Social and Behavioral Sciences, School of Public Health and Family Medicine, University of Cape Town, Cape Town 7925, South Africa; sofia.tclopes@gmail.com; 5 Department of Community Health, School of Public Health, University of Kinshasa, Kinshasa P.O. Box 11850, Democratic Republic of the Congo; dieudonne.mpunga@unikin.ac.cd

**Keywords:** women, empowerment, Kinshasa, PMA

## Abstract

Empowering women and promoting gender equality is crucial for accelerating sustainable development in fragile countries, including the Democratic Republic of Congo (DRC). However, there is scarce existing knowledge or understanding of the factors determining women’s empowerment in these contexts. We aimed to assess women’s empowerment and determine its associated factors in Kinshasa, DRC. We analyzed data from the 2021 Performance Monitoring Assessment (PMA) survey. A sample of 1365 women of childbearing age was retained for this study. Twenty empowerment items related to household decision-making, contraception use, and husband/partner influence were considered. We calculated the average women’s empowerment index (aWEI), identified the women’s empowerment variables using principal component analysis (PCA), and determined the associated factors for the first three principal components through the performance of multivariate binary logistic regression. In Kinshasa, the overall aWEI was estimated at 0.65. It was low for household decision-making (0.34) and high for husband/partner influence domains (0.93). Three principal components were identified and named, including the absence of threats, control of sexuality, and participation in decision-making. The factors associated with these components were having internet access, being in free union with a partner, being aged 40–49 years, and residing in a non-slum area. Increasing access to information would enable women in Kinshasa to make strategic decisions about their lives, benefiting themselves and others.

## 1. Introduction

In 2015, the United Nations (UN) adopted the Sustainable Development Goal 5 (SDG 5) of achieving gender equality and empowering all women and girls by 2030 to accelerate sustainable development [1]. Empowerment, known as the “expansion in people’s ability to make strategic life choices in a context where this ability was previously denied to them” [2], is at the heart of human development. Despite the considerable diversity in the literature on empowerment, women’s empowerment is a complex concept, and one of its features is decision-making power. According to Kabeer, women’s empowerment emerges from the combination of two essential components: resources and agency. Resources are essential preconditions for the empowerment process to take place; these include education and income, for example. Agency involves making a decision and acting according to that decision to achieve the desired outcome [3]. The process of empowerment is influenced by structural and contextual factors [4,5]. A woman’s ability to make decisions that influence her circumstances is an essential facet of empowerment and contributes to her general well-being.

Women’s empowerment is essential to global progress and an important asset for public development [1]. For the World Bank, empowered women are critical to economic growth, and the cost of gender disparities is a serious threat to development, especially in the poorest countries [6]. Empowered women can make important decisions and act for children’s health and their own health benefits [7], particularly in being able to make reproductive decisions including the use of contraceptives and when to have children, a right often limited or denied. The contribution of women to society’s development no longer needs to be demonstrated; countries that have empowered women to make meaningful life choices, act without constraints, and engage in any profession have witnessed significant development and growth [8]. However, gender gaps and disparities persist worldwide, particularly in fragile countries with limited resources [9].

Although the Democratic Republic of Congo (DRC) is committed to the 2030 Agenda, efforts to reverse gender disparities and empower women remain limited [10,11]. According to the Global Gender Gap Index, the country was ranked 140 out of 146 countries in 2023 [9]. Nationwide, women’s empowerment continues to be inadequate and characterized by significant gender disparity and gaps [6,12]. In 2022, a comprehensive report from the World Bank [6] updated the 2013 DHS rates [12] and indicated that only 15.1% of women had decision-making autonomy over their health, 21.1% regarding household expenses, and 16.6% for visits from relatives. The report identified “women’s lower access to certain key assets” as the driver of women’s empowerment regardless of its dimensions and life sectors, but advocated for an extensive exploration using robust and advanced analytical methodologies to determine the context-specific factors that could determine women’s empowerment [6].

Up to the date of this study, little was known about these factors in DRC. However, Castro Lopes et al. [13] in Mozambique and Abbas et al. [14] in Pakistan reported that age, level of education, women’s occupation, and wealth index impact women’s decision-making power. Belachew et al. [15] in Ethiopia and Sougou et al. [16] in Senegal also reported that age, educational level, religion, women’s residence in urban areas, and media exposure determine autonomy in the use of contraceptives. These factors cannot be extrapolated to Congolese women, who may have different social and environmental contexts.

DRC is large, multi-ethnic, and culturally grounded. It is listed among the poorest countries with high expected gender disparities. Kinshasa is a town with a multifaceted urban–rural context in DRC and encompasses almost all strata of this composition and gives a good picture of the country. Studying the factors that determine women’s empowerment in this specific context may provide input to a particular gender-oriented framework and policies for action in favor of Congolese women. We aimed to determine the factors associated with women’s empowerment through decision-making in Kinshasa in the DRC.

## 2. Materials and Methods

### 2.1. Study Design and Data Source

We conducted a secondary data analysis using data from the 2021 Performance Monitoring for Action (PMA2021) Survey conducted in Kinshasa between December 2021 and April 2022 [17]. The PMA project is an initiative of the USAID funded by the Bill & Melinda Gates Foundation to ensure the provision of quality data to guide family planning programs in 11 countries, including the DRC. It was designed as a longitudinal population-based cross-sectional panel, collecting information from the same women and households over time to track the progress of contraceptive use dynamics [18]. PMA surveys followed a rigorous population sampling process to ensure urban and rural representativeness for each province. In the DRC, the project focused on two provinces, Kinshasa and Kongo Central [17]. PMA surveys use a two-stage cluster sampling design with clusters selected through probability-proportional-to-size sampling, with 35 households selected randomly within each cluster. In each selected household, all women aged 15–49 were invited to participate. More details on the PMA methodology can be found at www.pmadata.org/data/survey-methodology (accessed on 17 January 2024).

Access to the 2021-onset Kinshasa PMA dataset was granted by the DRC’s Principal Investigator (P.A.) on request. The PMA project received ethical approval from the Institutional Review Boards (IRBs) at the Johns Hopkins Bloomberg School of Public Health (#14590) in November 2021 and the Kinshasa School of Public Health (KSPH) (#ESP/CE/159B/2021) in October 2021. Additionally, we obtained ethical approval from the KSPH-IRB (ID: ESP/CE/098/2024) in April 2024 to conduct this present secondary data analysis on the de-identified data of the 2021 Kinshasa PMA onset database.

**Figure 1:**
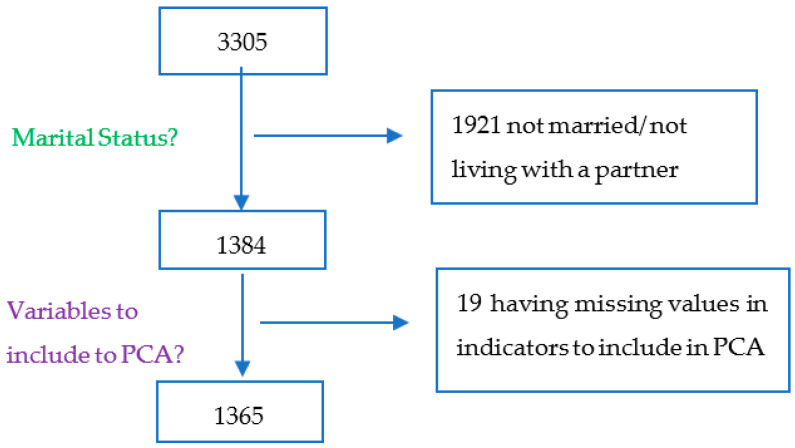
Data inclusion flowchart.

Of the 3305 women aged 15–49 included in the PMA dataset, we retained 1365 married/living with a partner woman in the present analysis (see Figure 1). We employed the data concerning the household level, such as the wealth indicator calculated using asset ownership, household size, and other socio-demographic characteristics, as well as the individual female data, such as measures of education; type of marriage; family planning access, choice, and use; women’s empowerment, etc.

### 2.2. Outcome Variables

#### 2.2.1. Empowerment Items in the Questionnaire

Women’s empowerment in the PMA study was studied following Kabeer and Mabsout’s framework [5,19] and benefited from contextual factors. The dataset and its questionnaire were screened, and 20 relevant questions/items related to women’s empowerment grouped under household decision-making, partner influence on having sex, and contraception utilization were identified. In this study, we used the definition of empowerment obtained from Kabeer on each item, where empowerment was defined as the capability to control and freely decide over one’s life [19].

#### 2.2.2. Computing the Average Women’s Empowerment Index (aWEI)

To report the prevalence of women’s empowerment, we computed: The average women’s empowerment index (aWEI) [20,21]. It is a reliable, standardized and composite index computed as averages scores from binary (1,0) items used to measure the potential of women to use their empowerment. It is expressed as a constant for all women and can be clustered to group items/individuals.

#### 2.2.3. Empowerment Item Reduction Using Principal Component Analysis

To identify associated factors, we needed to transform items into uncorrelated outcome variables for use in regression models. For that, we needed to use a technique that reduced 20 items into a small number of uncorrelated variables. We therefore performed principal component analysis (PCA).

Items from the dataset were re-coded into a 3-point scale (values of −1, 0, 1) where the highest value indicates a more significant level of women’s empowerment [8]. This approach distinguishes, in a particular area, women who were empowered, those who had some level of empowerment, and those who were utterly disempowered. Table S1 in the Supplementary Materials details the selected empowerment items from the questionnaire, the initial codes, and the transformations performed.

After re-coding, the 20 empowerment items were reduced to principal component score variables using PCA. PCA has been applied in studies on women’s empowerment to avoid the ad hoc estimation of summary scores in which each indicator has an equal contribution [8,22]. It allows the assessment of clustering patterns of empowerment indicators and the contribution (weight) of each component.

The first three significant components (with an eigenvalue above 1) were retained from the scree plot of the PCA results following the “Kaiser rule” [23,24]. An orthogonal varimax rotation was applied after confirming no correlation between the retained components, an essential criterion for this rotation type [25]. The Kaiser–Meyer–Olkin (KMO) measure of sampling adequacy was then applied to test how suitable the data were for PCA. The first three components represented the domains of women’s empowerment for Kinshasa and were named PC1 “safety and free from threats”, PC2 “control over sex/sexuality”, and PC3 “household decision making”. These were considered the outcome variables.

#### 2.2.4. Transformation of PC Score to Binary Outcomes

Each of the three component score variables was divided into quintiles from the most (5th quintile) to the least empowered women (1st quintile). The quintiles were then categorized into binary variables as the most vs. least empowered women (all groups below the 5th quintile). Each binary variable served as an outcome variable for the three logistic regression models.

### 2.3. Independent Variables

We considered household socio-economic, demographic, and individual behavioral variables from the dataset to relate to the three empowerment components. For the socio-economic variable, we considered the wealth index constructed on a PCA basis of household ownership asset quintiles (poorest, poor, middle, rich, richest). The demographic variables included age (less or equal to 19, 20–29, 30–39, 40–49 years), education (no education, primary, secondary, university), professional status (working in the last 7 days yes or no), marital status (married or living with partner), religion (Catholic, Protestant, Islam, Kimbanguist, Other Christian, African, Other religion), parity, household size, savings for the future, mobile money accounts, health insurance location (slum, non-slum),and area of residence. Behavioral variables included access to media (internet or not).

### 2.4. Data Analysis

The categorical variables are displayed in the tables and figures and expressed in percentages. The aWEI described the prevalence of women’s empowerment using key empowerment items from the PMA survey and by location (slum or non-slum). The PCA was used to reduce asset ownership to a wealth score variable categorized into quintile variables. We also used PCA to reduce the 20 women’s empowerment items to principal component (PC) variables. The first three PCs, with eigenvalues > 1, were transformed into binary variables to serve as outcome variables in the multiple logistic regression models. We estimated the association among socio-economic, socio-demographic, and behavioral characteristics and each component’s empowerment. Crude and adjusted odds ratios (OR) and respective 95% confidence intervals (95% CI) were calculated. STATA version 17 was used for all data analyses.

## 3. Results

### 3.1. Socio-Demographic Characteristics

A total of 1365 women were included in the present study. Table A1 in Appendix A shows their socio-demographic and behavioral characteristics. Women aged 30–39 years represented 40% of the total sample; meanwhile, the number of women aged 15–19 was lower, representing 1.8% of the total. Most women (91.5%) were educated at a secondary or tertiary level. Almost half of women (46.2%) were in a free union with a partner. Most women (79.1%) were residents of slum areas. Access to information on the internet was low, with only 35.3% of the women reporting having access to the internet.

### 3.2. Women Empowerment Domains and aWEI

**Figure A1:**
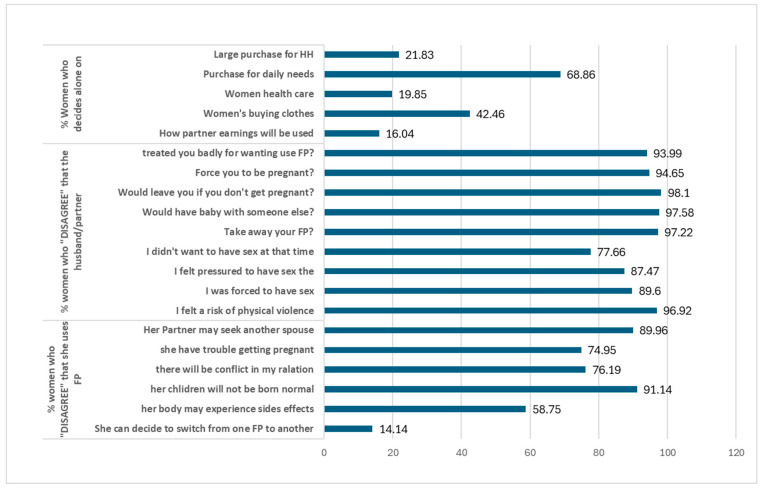
Empowerment items proportion for empowered women.

**Figure A2:**
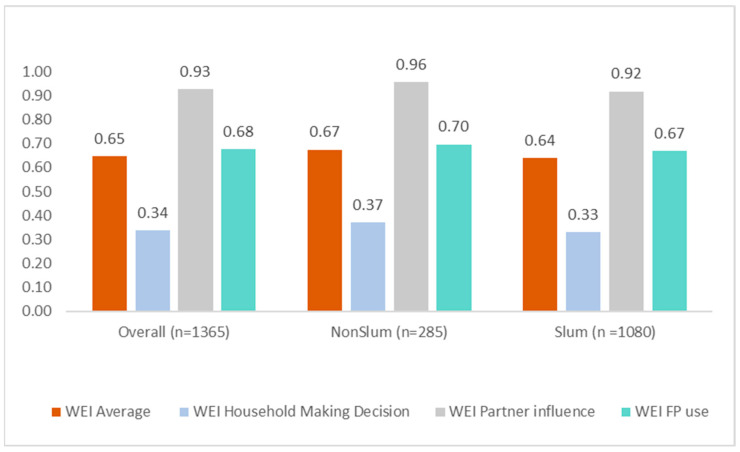
Average women’s empowerment index by domain and by slum EA.

Figure A1 displays the proportion of empowered women regarding each item of the empowerment domain; meanwhile, Figure A2 displays the aWEI for each domain of empowerment items and the overall aWEI across the location (slum or non-slum). The overall aWEI was estimated at 0.65, meaning that about 65% of women were able to achieve their empowerment potential. When stratifying this observation by domain, the household decision-making domain obtained a lower aWEI (0.34) and the partner influence domain obtained a higher aWEI (0.93).

### 3.3. Associated Factors to Women’s Empowerment

**Figure A3:**
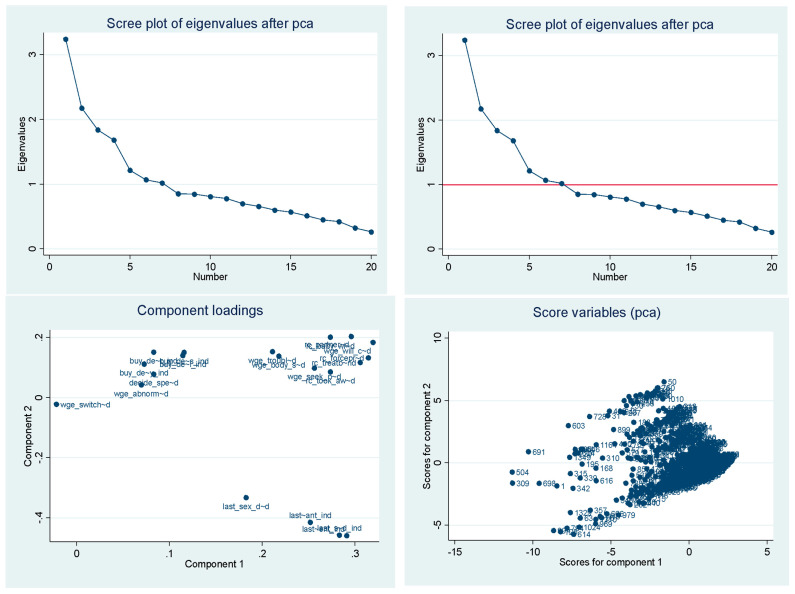
Scree plot.

Figure A3 displays the scree plot, which displays components across their respective eigen value. Details on these three retained components can be found in the Supplemental Materials.

Table A2 presents the crude and adjusted ORs for the association between empowerment domains and women’s socio-demographic, socio-economic, and behavioral characteristics.

Women’s age, level of education, marital status, parity, household size, having worked in the last seven days preceding the survey, having savings for the future, religion, having a mobile money account, their wealth quintile, and having health insurance were not associated with the empowerment PC1 of “safety and free from threats.” However, we noted a significant and positive association between access to the internet (aOR 1.83, 95% CI: 1.19, 2.83) and the component of safety and freedom from threats.

The empowerment PC2 “control over sex/sexuality” was significantly and positively associated with marital status (aOR: 1.96, 95% CI: 1.32, 2.89) and household size (aOR: 2.23, 95% CI: 1.05, 4.73). Women from 6–8 member households were 2.23 more likely to be empowered than those from 1–2 member households. No association was noted between age, level of education, parity, having worked in the last seven days preceding the survey, having savings for the future, religion, having a mobile money account, their wealth quintile, having health insurance, and access to the internet with control of sexuality.

Women aged 40–49 (aOR:2.02, 95% CI: 1.18, 3.46), marital status (aOR: 1.84, 95% CI: 1.23, 2.75), and internet access (aOR: 1.57, 95% CI: 1.02, 2.40) were significantly and positively associated with PC3 “household decision making”. We noted a significant, negative association between residence in slums (aOR: 0.66, 95% CI: 0.44, 0.99) and this empowerment PC. However, the level of education, parity, household size, having worked in the last seven days preceding the survey, having savings for the future, religion, having a mobile money account, their wealth quintile, and having health insurance were not associated with this PC.

## 4. Discussion

This study aimed to evaluate the degree of women’s empowerment and to determine the factors associated with women’s empowerment in Kinshasa in 2021. We calculated the average women’s empowerment index and estimated it at 0.65; this means that women are empowered to realize only 65% of their full potential and that the empowerment deficit amounts to almost 35%. This result is in line with the literature, which estimates the worldwide overall empowerment index at 0.607 [20,21]. The household decision-making obtained the lowest WEI after stratifying by domain, at 0.34. Also proved in the literature is that participation in decision-making has the lowest global WEI at 0.413 [20,21].

In this study, three domains of empowerment were highlighted using the PCA, notably the “absence of threats”, “control of sexuality”, and “participation in decision-making”. Empowerment is a multidimensional process occurring in different spheres of a woman’s life [26]. Therefore, each dimension (or domain) identified in this study, representing an area of a woman’s life where empowerment can take place, is determined by different factors. This is aligned with findings from other studies, Castro Lopes and Shimamoto [13,27]. The associated factors identified were age, marital status, household size, internet access, and residence in a slum. This is similar to the results of other studies carried out in Africa [8,13,28]. Our results show that the threat-free and decision-making domains of empowerment were influenced by internet access, with women having internet access being 1.83 times more likely not to suffer threats from their partners and 1.57 times more likely to make decisions than those who do not. Indeed, the literature also demonstrates a positive relationship between internet access and women’s empowerment, because this provides them with access to information. Women who have access to the internet are open to the wider world. Thus, they can know their rights as human beings, especially as women, and what the law says about domestic violence, which allows significant awareness of their personal value, updates them on changes taking place in different areas, and allows them to acquire knowledge, which enables consistent decision making. For the present study, access to the internet means “access to information on gender and rights and empowerment”. Therefore, access to information through the internet is an essential determinant for women’s empowerment. This conclusion is similar to the results of the research conducted by Castro Lopes and Aghazi [13,29].

Marital status and household size determine women’s empowerment in controlling their sexuality. Women in a free union are 1.96 times more likely to be autonomous about their sexuality than married women. This could be explained by the fact that, because they are not married to their partners, they are not under their guardianship; therefore, they can freely decide about their sexuality. In addition, on a legal level, they do not have duties toward their partners; hence, they can freely decide on their sexuality [30,31]. we advance that awareness campaigns involving men should be put in place to work on the beliefs and norms that put men in a position of power over women when married.

Our results suggest a positive and significant relationship between household size and empowerment regarding sexual control; women living in households with sizes ranging from 6 to 8 members are 2.23 times more likely to be empowered. Furthermore, a positive relationship was observed between women with 8 or more births and increased chance of empowerment in this domain. This might be explained by the fact that a larger household size increases the domestic burden, both financially and in terms of housework, which could lead to an imbalance for women; being assumed to be responsible for housework, they are able to devote little time to other activities and find themselves to be dependent. Other reasons include women wanting to ensure to have the right socioeconomic conditions to raise their children as well as to reduce the chances of poor health outcomes for themselves, as reported in a study conducted in Mozambique [32]. The realization of all of the burdens that impinge on their freedom, on their living conditions and on their health can lead them to take control of their sexuality. Similar results were demonstrated in the studies of Kegnide and Soharwardi [33,34]. Nevertheless, Aghazi and Akram [29,35] found a negative relationship between household size and women’s empowerment. This inconsistency can be explained by the different sample sizes and family systems. In fact, in the regions of Pakistan and Ethiopia, households are made up of several small families, which means women have little decision-making power, which is not the case in the city of Kinshasa.

Our results show that decision-making is influenced by being in the age group of 40–49. Women aged 40–49 are more likely to be autonomous in making decisions, whether for their health or within the household, than younger women. Interestingly, this relationship was not observed with the domain control over sex/sexuality. This could be explained by the fact that older women have more experience in several areas of life and gain awareness and trust from their partners over time; these results confirm those identified in the work of Abbas, Akram, and Sougou [14,16,35].

However, when it comes to reproductive decisions and sexuality, the number of births/pregnancies seems to be more influential. Other studies also suggest that access to information through health providers or other means supports women to have more control over their sexual and reproductive lives. Marital status also influences decision-making. Women in a free union are 1.84 times more likely to make decisions alone than married women. This result can be explained by the fact that the DRC is traditionally a patriarchal society in which the household decision-makers are men. Women are the responsibility of their husbands, who can, therefore, make decisions for them. However, women in free unions are not under the control of their husbands and are more autonomous in making their own decisions [36,37,38].

The results of our study suggest that residence in a slum makes women less likely to be independent than those living in a non-slum. Indeed, women living in slums live in precarious conditions; they are not exposed to the media daily, which limits their access to information and their knowledge of the world, which are essential drivers of empowerment. They also experience difficulties in income-generating activities, making them dependent on their spouse, thus limiting their decision-making power [39].

The present study has strengths and weaknesses. The study’s strength lies in the fact that it is the first to identify the factors associated with women’s empowerment in Kinshasa, which is necessary to complete a study on this subject. This study used a large sample of women aged 15–49 from the PMA survey, allowing for the target population’s representativeness. The PMA survey collected and measured three dimensions of women’s empowerment, and this secondary analysis used PCA methods to account for the individual effects of items and avoid the ad hoc estimation of summary scores in which each indicator has an equal contribution. The clustering patterns from more than one dimension and 20-item empowerment indicators have contributed to the strengths of the present study. The logistic regression model used to determine associated factors was suitable for controlling confounding factors and checking for interactions.

As for the weaknesses of the study, it should be noted that the design of the PMA survey was cross-sectional, which means that it is impossible to establish a cause-and-effect relationship. To achieve this, a longitudinal analysis would be necessary, given that women’s empowerment is a process of acquisition of equal power. In addition, information on women’s empowerment in the PMA survey only targeted married women and those in union with a partner; other categories were not considered. Another weakness of this study is that it could present a classification bias, given that it classified women into two groups, the most empowered (as the fifth quintile) and less empowered women (from the first to the fourth quintiles), using the quintile variable. Another classification might find other proportions, but the classification we considered supports the scenario of high empower rank difference and has been used in the literature. This study was limited to the quantitative aspect based on the available data, which made it possible to define the different areas of empowerment; therefore, we believe that additional, more in-depth research, integrating a qualitative component, could elicit additional information concerning the indicators of women’s empowerment. Comparing the two approaches, the aWEI estimation sums variables that seem to be related and are suitable for prevalence, while PCA allows better clustering of variables for accurate estimation of emergent women’s empowerment by domain and their associated factors by logistic regression. The two approaches were then complementary and critical to the objective of this study:

## 5. Conclusions

Access to information and communication literacy broadens the outlook and builds awareness. Women with knowledge and good information about their rights, roles, and gender equity can make meaningful life decisions, act without constraints, and contribute to the significant development of their family growth, economy, and health. Enhancing women’s access to information and communication (the internet) has the potential to help women reach different dimensions of their empowerment. Making resources available for them at home, at their place of business, or in a nearby area can increase the empowerment average. Our results also advocate that awareness campaigns should involve men, recognizing their position of power on the beliefs and social norms of married women.

## Data Availability

The data used for this publication and the DO file can be made available to the corresponding author upon reasonable request.

## Supplementary Materials

The following supporting information can be downloaded at: https://www.mdpi.com/article/10.3390/ijerph21070943/s1, Table S1: Transformation and recoding of empowerment items.

## Appendix A

**Table A1:** Distribution of women’s socio-demographic, socio-economic, and behavioral characteristics.

	N	%
**Age (years)**		
15–19	25	1.83
20–29	454	33.26
30–39	546	40.00
40–49	340	24.91
**Total**	**1365**	**100.00**
**Education**		
Never	4	0.29
Primary	110	8.06
Secondary	971	71.14
Tertiary	280	20.51
**Total**	**1365**	**100.00**
**Marital status**		
Married	734	53.77
Not married but living with a partner	631	46.23
**Total**	**1365**	**100.00**
**Ever birth**		
No	76	5.57
Yes	1289	94.43
**Total**	**1365**	**100.00**
**Birth events**		
Undelivered or first	255	19.78
2–4 deliveries	732	56.79
5–7 deliveries	263	20.40
8+ deliveries	39	3.03
**Total**	**1289**	**100.00**
**Household size**		
1–2 members	640	66.53
3–5 members	270	28.07
6–8 members	41	4.26
9+ members	11	1.14
**Total**	**962**	**100.0**
**Work in the last seven days**		
No	638	46.74
Yes	726	53.19
**Total**	**1364**	**100.00**
**Have savings for the future**		
No	960	70.33
Yes	405	29.67
**Total**	**1365**	**100.00**
**Have mobile money accounts**		
No	883	64.69
Yes	482	35.31
**Total**	**1365**	**100.00**
**Internet access**		
No	883	64.69
Yes	482	35.31
**Total**	**1365**	**100.00**
**Have insurance health or be a member of a mutual health organization**		
No	1175	86.08
Yes	190	13.92
**Total**	**1365**	**100.00**
**Religion**		
Catholic	170	13.01
Protestant	96	7.35
Other Christian	853	65.26
Kimbanguiste	47	3.60
Islamic	22	1.68
African (bundu, vuvamu, animist)	14	1.07
Other religion	105	8.03
**Total**	**1307**	**100.00**
**Slum EA**		
Non-slum	285	20.88
Slum	1080	79.12
**Total**	**1365**	**100.00**
**Wealth quintile**		
Lowest	223	23.16
Lower	194	20.15
Middle	201	20.87
Higher	178	18.48
Highest	167	17.34
**Total**	**963**	**100.00**

**Table A2:** Factors Associated with Women’s Empowerment in Kinshasa in 2021.

	PC1 Named “*Safety and Free from Threats*”		PC2 Named “*Control over Sex/Sexuality*”		PC3 Named *“Household Decision Making*”	
	cOR (95% CI)	aOR (95% CI)	cOR (95% CI)	aOR (95% CI)	cOR (95% CI)	aOR (95% CI)
**Age (years)**						
15–29	1	1	1	1	1	1
30–39	1.54 (1.12, 2.12)	1.52 (0.93, 2.50)	0.97 (0.71, 1.32)	0.91 (0.58, 1.43)	1.79 (1.29, 2.48)	1.39 (0.87, 2.20)
40–45	1.66 (1.16, 2.36)	1.76 (0.99, 3.12)	1.27 (0.91, 1.79)	1.35 (0.80, 2.29)	1.82 (1.28, 2.62)	2.02 (1.18, 3.46)
**Education**						
Never or Primary	1	1	1	1	1	1
Secondary	1.20 (0.69, 2.07)	0.84 (0.41, 1.71)	0.89 (0.57, 1.38)	1.21 (0.67, 2.18)	1.12 (0.67, 1.89)	0.94 (0.49, 1.79)
Tertiary	2.57 (1.45, 4.57)	1.16 (0.49, 2.72)	0.33 (0.18, 0.59)	0.93 (0.41, 2.11)	1.83 (1.05, 3.19)	1.42 (0.64, 3.17)
**Marital status**						
Married	1	1	1	1	1	1
Living with a partner	0.58 (0.44, 0.76)	1.02 (0.67, 1.56)	1.67 (1.28, 2.18)	1.96 (1.32, 2.89)	0.97 (0.74, 1.26)	1.84 (1.23, 2.75)
**Birth events**						
Undelivered or first	1	1	1	1	1	1
2–4 deliveries	1.12 (0.78, 1.59)	0.98 (0.59, 1,63)	1.22 (0.84, 1.78)	1.56 (0.92, 2.63)	1.39 (0.96, 2.01)	1.33 (0.80, 2.19)
5–7 deliveries	0.82 (0.53, 1.29)	0.77 (0.39, 1.48)	1.66 (1.08, 2.57)	1.64 (0.86, 3.11)	0.91 (0.57, 1.45)	0.81 (0.42, 1.56)
8+ deliveries	1.06 (0.46, 2.44)	1.01 (0.32, 3.21)	2.84 (1.36, 5.91)	3.83 (1.42, 10.30)	1.08 (0.45, 2.60)	1.66 (0.58, 4.76)
**Household size**						
1–2 members	1	1	1	1	1	1
3–5 members	1.11 (0.78, 1.58)	1.11 (0.75, 1.65)	1.09 (0.78, 1.54)	1.31 (0.89, 1.91)	1.15 (0.82, 1.62)	1.24 (0.85, 1.81)
6–8 members	1.19 (0.56, 2.57)	1.37 (0.61, 3.11)	1.52 (0.76, 3.06)	2.23 (1.05, 4.73)	1.22 (0.58, 2.55)	1.35 (0.60, 3.04)
9+ members	0.42 (0.05, 3.35)	0.39 (0.05, 3.51)	0.82 (0.17, 3.82)	0.35 (0.04, 3.08)	0.84 (0.18, 3.93)	0.43 (0.05, 3.62)
**Work in the last seven days**						
No	1	1	1	1	1	1
Yes		1.04 (0.72, 1.52)		0.81 (0.57, 1,15)		0.84 (0.59, 1.19)
**Have savings for the future**						
No	1	1	1	1	1	1
Yes	1.11 (0.84, 1.48)	1.09 (0.73, 1.63)	0.71 (0.52, 0.96)	0.87 (0.58, 1.31)	1.27 (0.96, 1.69)	1.27 (0.86, 1.86)
**Have mobile money accounts**						
No	1	1	1	1	1	1
Yes	2.06 (1.57, 2.69)	1.42 (0.95, 2.12)	0.68 (0.51, 0.91)	0.89 (0.59, 1.35)	1.56 (1.19, 2.05)	1.19 (0.81, 1.77)
**Internet access (exposure to media)**						
No	1	1	1	1	1	1
Yes	2.18 (1.67, 2.86)	1.83 (1.19, 2.83)	0.68 (0.51, 0.91)	1.11 (0.71, 1.73)	1.66 (1.26, 2.17)	1.57 (1.02, 2.40)
**Have insurance health or be a member of a mutual health organization**						
No	1	1	1	1	1	1
Yes	1.04 (0.71, 1.52)	0.73 (0.43, 1.23)	0.69 (0.45, 1.05)	0.92 (0.54, 1.59)	1.34 (0.93, 1.93)	0.91 (0.55, 1.50)
**Religion**						
Catholic	1	1	1	1	1	1
Protestant	0.91 (0.49, 1.68)	0.85 (0.41, 1.76)	1.51 (0.85, 2.69)	1.32 (0.67, 2.60)	1.26 (0.69, 2.29)	1.17 (0.59, 2.34)
Other Christian	0.85 (0.57, 1.27)	0.79 (0.48, 1.29)	0.95 (0.63, 1.42)	0.90 (0.55, 1.47)	0.97 (0.64, 1.47)	0.98 (0.60, 1.61)
Kimbanguiste	0.24 (0.07, 0.81)	0.28 (0.06, 1.31)	0.68 (0.28, 1.64)	0.65 (0.25, 1.70)	0.95 (0.42, 2.15)	0.79 (0.29, 2.18)
Islamic	1.02 (0.35, 2.95)	1.77 (0.52, 6.01)			0.40 (0.09, 1.79)	0.54 (0.11, 2.72)
African (bundu, vuvamu, animist)	1.93 (0.61, 6.10)	4.64 (0.59, 36.47)	1.05 (0.28, 3.98)	1.02 (0.09, 11.13)	1.60 (0.47, 5.41)	4.69 (0.58, 38.04)
Other religion	1.09 (0.61, 1.93)	1.16 (0.57, 2.38)	0.85 (0.46, 1.58)	0.85 (0.42, 1.74)	1.19 (0.66, 2.14)	1.02 (0.49, 2.08)
**Slum EA**						
Non-slum	1	1	1	1	1	1
Slum	0.59 (0.44, 0.81)	0.77 (0.51, 1.16)	1.29 (0.92, 1.83)	1.05 (0.67, 1.62)	0.59 (0.44, 0.81)	0.66 (0.44, 0.99)
**Wealth quintile**						
Lowest	1	1	1	1	1	1
Lower	1.68 (0.96, 2.94)	1.49 (0.80, 2.81)	0.61 (0.38, 0.96)	0.75 (0.45, 1.26)	0.94 (0.57, 1.56)	0.93 (0.53, 1.63)
Middle	1.49 (0.85, 2.63)	1.12 (0.59, 2.14)	0.72 (0.46, 1.11)	0.85 (0.51, 1.41)	1.03 (0.63, 1.69)	0.89 (0.51, 1.56)
Higher	2.76 (1.62, 4.71)	1.59 (0.82, 3.07)	0.61 (0.38, 0.97)	0.79 (0.44, 1.42)	1.46 (0.90, 2.36)	0.97 (0.53, 1.78)
Highest	3.48 (2.05, 5.92)	1.40 (0.69, 2.87)	0.57 (0.35, 0.92)	0.89 (0.46, 1.75)	2.01 (1.25, 3.22)	1.08 (0.56, 2.08)
