# Supplementary material for: Women’s Empowerment and Associated Factors in Kinshasa, Democratic Republic of Congo: A Secondary Data Analysis of the Performance Monitoring Assessment Survey
Source: Int J Environ Res Public Health. 2024 Jul 19;21(7):943. doi: 10.3390/ijerph21070943 (PMC11276809; doi:10.3390/ijerph21070943)
Supplement: Supplementary file 1 [file ijerph-21-00943-s001.zip › ijerph-3056844-supplementary.pdf]

Supplementary Materials

Women's Empowerment and Associated Factors in Kinshasa, Democratic Republic of Congo: A Secondary Data Analysis of the Performance Monitoring Assessment Survey

Table S1. Transformation and recoding of empowerment items

Household Decision-Making				
**item**	**Questions**	**Codes**	**Recording**	**Scale**
decision_medical	Who usually decides on women's health care?	Respondent alone=1; husband/partner alone=2 Respondent and husband/partner jointly=3; Someone else=4	Respondent alone =1; Jointly= 0; Partner or other alone= -1	Nominale
decision_major	Who usually makes decisions about making large household purchases?	Respondent alone=1; husband/partner alone=2 Respondent and husband/partner jointly=3; Someone else=4	Respondent alone =1; Jointly= 0; Partner or other alone= -1	Nominale
decision_daily	Who usually makes decisions about making household purchases for daily needs?	Respondent alone=1; husband/partner alone=2 Respondent and husband/partner jointly=3; Someone else=4	Respondent alone =1; Jointly= 0; Partner or other alone= -1	Nominale
decision_clothes	Who usually makes decisions about buying women's clothes?	Respondent alone=1; husband/partner alone=2 Respondent and husband/partner jointly=3; Someone else=4	Respondent alone =1; Jointly= 0; Partner or other alone= -1	Nominale
Decide_spending_partner	Who usually makes decisions about how husband's earnings will be used?	Respondent alone=1; husband/partner alone=2 Respondent and	Respondent alone =1; Jointly= 0; Partner or other alone= -1	Nominale

		husband/partner jointly=3; Someone else=4		
**Partner/husband influence**				
**item**	**Questions**	**Codes**	**Recording**	**Scale**
partner_fp_sad_12m	Has your husband/partner made you feel bad or treated you badly for wanting to use a FP method to delay or prevent pregnancy?	No=0 ; Yes=1	No=1 ; Don't Know=0 ; Yes=-1	Nominale
partner_force_sex_12m	Has your husband/partner Tried to force or pressure you to become pregnant?	No=0 ; Yes=1	No=1 ; Don't Know=0 ; Yes=-1	Nominale
partner_abandon_12m	Has your husband/partner Said he would leave you if you did not get pregnant?	No=0 ; Yes=1	No=1 ; Don't Know=0 ; Yes=-1	Nominale
partner_cheat_12m	Has your husband/partner Told you he would have a baby with someone else if you did not get pregnant?	No=0 ; Yes=1	No=1 ; Don't Know=0 ; Yes=-1	Nominale
partner_fp_take_12m	Taken away your family planning or kept you from going to the clinic to get family planning?	No=0 ; Yes=1	No=1 ; Don't Know=0 ; Yes=-1	Nominale
last_sex_not_want	At the last time you had sex, did you want to have it at that time?	No=0 ; Yes=1	No=1 ; Don't Know=0 ; Yes=-1	Nominale
last_sex_pressured	At the last time you had sex, were you felt pressured by your husband / partner to have sex then?	No=0 ; Yes=1	No=1 ; Don't Know=0 ; Yes=-1	Nominale
last_sex_not_consent	At the last time you had sex, do you	No=0 ; Yes=1	No=1 ; Don't Know=0 ; Yes=-1	Nominale

	consent (was forced) to have sex then?			
last_sex_duress	At the last time you had sex, have you felt at risk of physical violence if you declined to have sex at that time?	No=0 ; Yes=1	No=1 ; Don't Know=0 ; Yes=-1	Nominale
**Contraception utilization**				
**item**	**Questions**	**Codes**	**Recoding**	**Scale**
Seek_partner	If I use family planning, my husband/partner may seek another sexual partner	Strongly disagree =1; Disagree= 2; Neither agree nor disagree= 3; Agree=4; Strongly agree= 5; Do not know=-88; No response=-99	Strongly disagree or disagree=1; Neither agree or don't know=0; Agree or strongly agree=-1	Nominale
trouble_preg	If I use family planning, I may have trouble getting pregnant the next time I want to	Strongly disagree =1; Disagree= 2; Neither agree nor disagree= 3; Agree=4; Strongly agree= 5; Do not know=-88; No response=-99	Strongly disagree or disagree=1; Neither agree or don't know=0; Agree or strongly agree=-1	Nominale
will_conflict	There will be conflict in my relationship/marriag e if I use family planning	Strongly disagree =1; Disagree= 2; Neither agree nor disagree= 3; Agree=4; Strongly agree= 5; Do not know=-88; No response=-99	Strongly disagree or disagree=1; Neither agree or don't know=0; Agree or strongly agree=-1	Nominale
abnormal_birth	If I use family planning, my children may not be born normal	Strongly disagree =1; Disagree= 2; Neither agree nor disagree= 3; Agree=4; Strongly agree= 5; Do not know=-88; No response=-99	Strongly disagree or disagree=1; Neither agree or don't know=0; Agree or strongly agree=-1	Nominale
body_side_effects	If I use family planning, my body may experience side effects that will disrupt my relations	Strongly disagree =1; Disagree= 2; Neither agree nor disagree= 3; Agree=4; Strongly	Strongly disagree or disagree=1; Neither agree or don't know=0;	Nominale

	with my husband/partner	agree= 5; Do not know=-88; No response=-99	Agree or strongly agree=-1	
switch_fp	I can decide to switch from one family planning method to another if I want to	Strongly disagree =1; Disagree= 2; Neither agree nor disagree= 3; Agree=4; Strongly agree= 5; Do not know=-88; No response=-99	Strongly disagree or disagree=1; Neither agree or don’t know=0; Agree or strongly agree=-1	Nominale
